# Relationship between high intra-abdominal pressure and compliance of the pelvic floor support system in women without pelvic organ prolapse: A finite element analysis

**DOI:** 10.3389/fmed.2022.820016

**Published:** 2022-08-08

**Authors:** Xiaode Liu, Qiguo Rong, Yanan Liu, Jianliu Wang, Bing Xie, Shuang Ren

**Affiliations:** ^1^X Lab, The Second Academy of China Aerospace Science and Industry Corporation, Beijing, China; ^2^Beijing Key Laboratory of Sports Injuries, Department of Sports Medicine, Institute of Sports Medicine of Peking University, Peking University Third Hospital, Beijing, China; ^3^Department of Mechanics and Engineering Science, College of Engineering, Peking University, Beijing, China; ^4^Department of Obstetrics and Gynecology, Dongping County People’s Hospital, Taian, China; ^5^Department of Obstetrics and Gynecology, Peking University People’s Hospital, Peking University, Beijing, China; ^6^Beijing Key Laboratory of Female Pelvic Floor Disorders, The Research Center of Female Pelvic Floor Disorder Disease of Peking University, Beijing, China; ^7^Joint International Research Center of Translational and Clinical Research, Beijing, China

**Keywords:** pelvic floor support system, finite element method, prolapse, compliance, healthy woman

## Abstract

Previous studies mainly focused on the relationship between the size of the prolapse and injury to the supporting tissues, but the strain and stress distributions of the supporting tissues as well as high-risk areas of injury are still unknown. To further investigate the effect of supporting tissues on organs and the interactions between organs, this study focused on the relationship between high intra-abdominal pressure and the compliance of the pelvic floor support system in a normal woman without pelvic organ prolapse (POP), using a finite element model of the whole pelvic support system. A healthy female volunteer (55 years old) was scanned using magnetic resonance imaging (MRI) during rest and Valsalva maneuver. According to the pelvic structure contours traced by a gynecologist and anatomic details measured from dynamic MRI, a finite element model of the whole pelvic support system was established, including the uterus, vagina with cavity, cardinal and uterosacral ligaments, levator ani muscle, rectum, bladder, perineal body, pelvis, and obturator internus and coccygeal muscles. This model was imported into ANSYS software, and an implicit iterative method was employed to simulate the biomechanical response with increasing intra-abdominal pressure. Stress and strain distributions of the vaginal wall showed that the posterior wall was more stable than the anterior wall under high intra-abdominal pressure. Displacement at the top of the vagina was larger than that at the bottom, especially in the anterior–posterior direction. These results imply potential injury areas with high intra-abdominal pressure in non-prolapsed women, and provide insight into clinical managements for the prevention and surgical repair plans of POP.

## Introduction

Pelvic organ prolapse (POP) is a common problem for elder women specifically for the females after postmenopause and is generally associated with defects or injuries of the pelvic floor support system (1). Half of all parous women have experienced POP, and 10–20% lifetime risk need surgical care (2). Increased intra-abdominal pressure, such as with loaded walking, coughing, sneezing, squatting, defecating, and bending, is an important independent risk factor for POP (3). Constipation as well as obesity can also induce chronic high intra-abdominal pressure (2).

A number of studies (4–6) have simulated anterior and posterior vaginal wall prolapse under high intra-abdominal pressures. These studies showed that combined injury to the levator ani muscle and the vaginal apex results in anterior vaginal wall prolapse, and combined injury to the levator ani muscle and posterior supporting tissues leads to posterior vaginal wall prolapse. These findings were of great significance for exploring the mechanism of vaginal prolapse. However, these studies mainly focused on the relationship between the size of the prolapse and injury to the supporting tissues, and neglected the strain and stress distributions of the supporting tissues as well as high-risk areas of injury. Another important factor for prolapse is the effect of supporting tissues on organs and the interactions between organs. Few studies have reported these two crucial factors in healthy people.

The objective of this study was to simulate the pelvic visceral mechanical response of non-prolapsed women under high intra-abdominal pressure using a three-dimensional (3D) finite element model (FEM) of the pelvic floor support system. The model was established using ANSYS software (ANSYS, Houston, TX, United States), and the anatomy of the single volunteer subject was obtained by magnetic resonance imaging (MRI). Vaginal wall displacement and the distributions of stress and strain in the supporting tissues were calculated, and possible initial damage points in the supporting tissue and the relationship between intra-abdominal pressure and pelvic floor visceral displacement were studied.

## Materials and methods

### Reconstruction of 3D FEM

**FIGURE 1:**
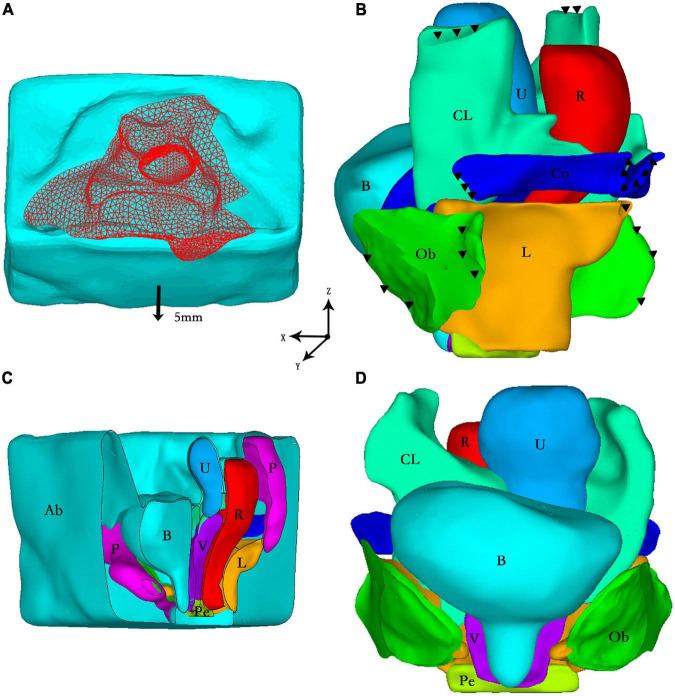
**(A)** Loading conditions: vertical peritoneal uniform pressure load (red) and forward displacement (arrow); **(B)** Boundary conditions: fixed constraint (black triangle) in the side view of the model; **(C)** The sectional view of the full model; **(D)** The front view of organs and supporting tissues. Ab, abdominal cavity; B, bladder; CL, cardinal ligaments; U, uterus; R, rectum; Co, coccygeus; Ob, obturator internus; L, levator ani muscle; P, pelvis; Pe, perineal body; V, vagina.

One asymptomatic and physical examination confirmed healthy female volunteer (55 years old, BMI: 20.96 kg/m^2^) with no previous pelvic surgery was recruited. The subject signed informed consent for inclusion in this institutional review board-approved study. This is a 50th demographic percentile subject from a IRB-approved case-control mechanistic cohort study at the Peking University People’s Hospital comparing women with anterior vaginal wall prolapse with normal asymptomatic women (Institutional Review Board HUM00012823). Axial, sagittal, and coronal MRI images were acquired while the subject was in the supine position during rest T2 and Valsalva using a 3.0-T GE scanner (Discovery MR750 3.0 T; GE Healthcare, Milwaukee, WI, United States) with a 32-channel, torso phased-array coil. The pelvic structure contours were traced with the following parameters: TR/TE 3,000/102–108 ms; field of view 26–28 cm; slice thickness 4 mm interleaved; gap 1 mm; acquisitions 2, and 90 continuous images were obtained. The MRI images were then imported into the medical image processing software Mimics 10.01 (Materialise Inc., Leuven, Belgium) for 3D calculations and reconstruction by an experienced urogynecologist. The 3D model was segmented into anatomic structures, including pelvic bones, bladder, urethra, vagina, uterus, rectum, obturator internus, cardinal ligaments, uterosacral ligaments, and five branches of the levator ani muscles. Then every single structure above was exported as STL files and imported into Geomagic Studio software (version 12.0; Geomagic, Inc., Morrisville, NC, United States) for more detailed pre-processing, such as smoothing and positioning. Finally, the entire model was imported into ANSYS software version 14.0 (Houston, TX, United States) for the study. The contacts between each supporting tissues and organs were established by sharing contact faces through Boolean operations. The finite element model was meshed with 10-node tetrahedral element and it contained 503368 elements and 696101 nodes. The sectional view of the full model and the front view of organs and supporting tissues were shown in Figures 1C,D, respectively. The detailed description of FEM was described and validated in our previous publication (7).

### Material properties

All material properties were considered linear elastic to simplify the numerical simulations. The elastic modulus of the supporting was based on perineal body data derived from *in vivo* measurements of healthy nulliparous women (8). Uniaxial tension data from cadaveric specimens (9, 10) was used for the elastic modulus of the apex ligaments. Since there are no existing data describing the fascial properties in the literature, we assumed that the elastic modulus of the fascia was half that of the perineal body, and that the elastic modulus of the abdominal cavity was half that of the fascia. Poisson’s ratio for the fascia and abdominal cavity was 0.3 and 0.49, respectively.

The mechanical properties of the vagina in previous studies were mostly measured *in vitro*, and the data varied from one study to another. Considering that the properties of connective tissue in the abdominal cavity *in vivo* are quite different from those *in vitro*, and that the vagina was similar to fascia based on the clinical experience, we chose 0.015 MPa instead of using the data in the literature, for this study. The data for the bladder and rectum were scaled up to the same magnitude as that of the vagina according to measurements in previous studies. Regarding the uterus, few studies have reported uterine material properties in nulliparous women. Therefore, we used the characteristics of uterine samples from pregnant women for this analysis. At high intra-abdominal pressure, contraction of the muscles in the pelvic wall (including the peritoneum) leads to higher elastic modulus of tissues (11); thus, the pelvic wall had the same properties as the attached muscles. All of the material parameters mentioned above are presented in Table 1.

**TABLE 1:** Material parameters of pelvic tissue.

Tissue	Poisson ratio (μ)	Modulus of elasticity (E) MPa
Levator ani muscle (18, 24)	0.3	0.8654
Posterior ligamentous complex (9)	0.3	0.3251
Perineal body (8)	0.3	0.0289
Vagina (22)	0.3	0.015
Uterus (25)	0.49	0.486
Bladder (26)	0.49	0.01519
Rectum (26)	0.45	0.01142
Abdominal cavity	0.49	0.0072
Fascia (18)	0.3	0.0144
Coccygeal muscle, obturator muscles (11, 18), and peritoneal wall (26)	0.3	13.37

### Boundary conditions

It was assumed that the pelvis was fixed, and that all nodes in the pelvis were fully constrained. The ligaments and muscles could not move relative to the pelvis, as shown in Figure 1B. Regarding the load conditions, the intra-abdominal pressure during the Valsalva maneuver mentioned in the literature (4, 5) was approximately 70–168 cm H_2_O. In this study, we loaded with a uniform 100 cm H_2_O (0.01 MPa) intra-abdominal pressure on the peritoneum to represent the situation under higher intra-abdominal pressure. When the volunteer performed the Valsalva maneuver, the abdominal wall moved 5 mm forward, which was documented via dynamic MRI. Thus, the abdominal wall in this model involved a 5-mm forward displacement (Figure 1A). We used the Newton-Raphson method to perform the analysis until convergence was obtained. It took approximately 30 mins to complete the simulation on a computer with an Intel^®^ Core™ i7-4790 processor (IBM Corp., Armonk, NY, United States) with 3.60 GHz CPU and 32.0 G RAM running Windows 7 professional version (Microsoft Corp., Redmond, WA, United States).

## Results

### Compliance

Backward and downward displacement of the vagina were observed with increasing intra-abdominal pressure under normal pelvic support. Displacement of the top of the vagina was larger compared with that of the bottom of the vagina. The results were in good agreement with the vaginal displacement observed in dynamic MRI, as shown in Table 2.

**TABLE 2:** Comparison of vaginal displacement under high intra-abdominal pressure.

	Displacement from MRI (mm)	Displacement from simulation (mm)
Proximal point of anterior vaginal wall in y	10.24	5.29
Proximal point of anterior vaginal wall in z	7.81	8.1
Distal point of anterior vaginal wall in y	−2.9	−0.3
Distal point of anterior vaginal wall in z	5.9	5.6

For the anterior and posterior vaginal walls, 13 nodes were selected from the top to the bottom. The most distal edge of the cervix was set as the C point, and the compliance of the anterior vaginal wall, posterior vaginal wall, and the C point was explored.

**FIGURE 2:**
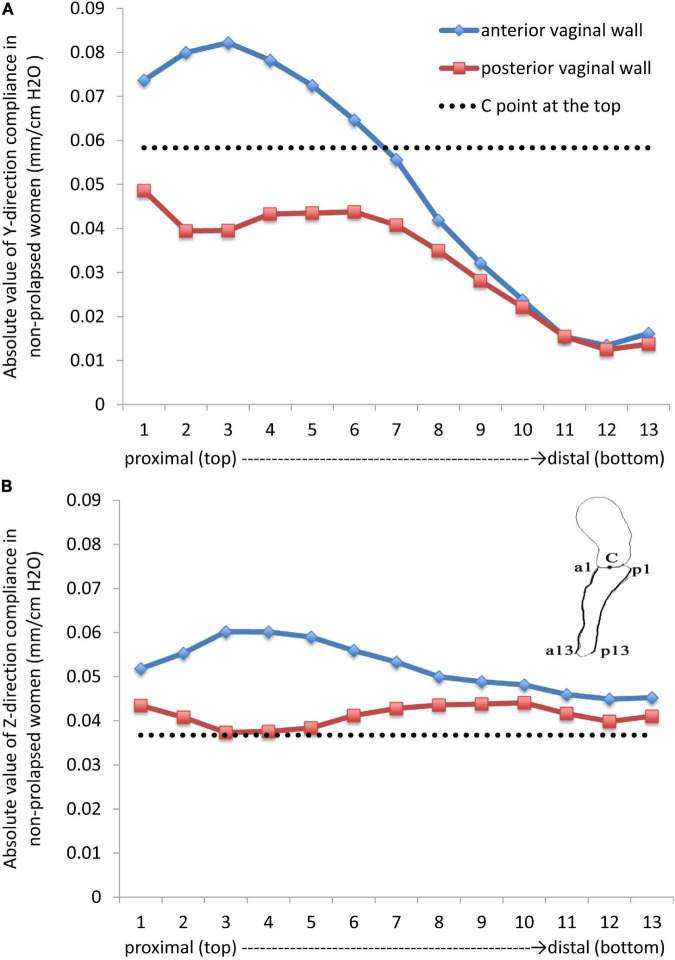
Absolute values in the **(A)** (forward and backward) Y direction and **(B)** (vertical) Z direction compliances in non-prolapsed women.

The vertical, and forward and backward directions were defined as the Z direction and Y direction in a coordinate system, respectively. The largest vertical displacement occurred at the top of the bladder, while the smallest displacement was detected at the side walls of the uterus and vagina and at the bottom of the bladder. Figure 2 shows the compliance of the anterior vaginal wall and the posterior vaginal wall, with 1-cm H_2_O increments of intra-abdominal pressure. Absolute values were used for analysis as all the displacements were negative (Figure 2). Regarding the Z direction, the compliance of the anterior vaginal wall (Figure 2, in blue) showed a downward trend from the top to the bottom, which was consistent with the clinical observation. The compliance of the anterior vaginal wall was slightly higher than that of the posterior vaginal wall. In the upper third of the vagina, the compliance of the anterior and posterior vaginal walls showed the most significant difference. As the C point of the cervix was supported by the cardinal and uterosacral ligaments complex, its downward compliance was lower than that of the vaginal wall (Figure 2A). The Y direction compliance of the vaginal wall decreased gradually from the top to the bottom, and its descending speed was higher than that in the Z direction (Figure 2). The Y direction displacement compliance of the anterior vaginal wall was higher than the posterior vaginal wall, similar to the Z direction.

### Distributions of stress and strain in supporting tissues of the pelvic floor

**FIGURE 3:**
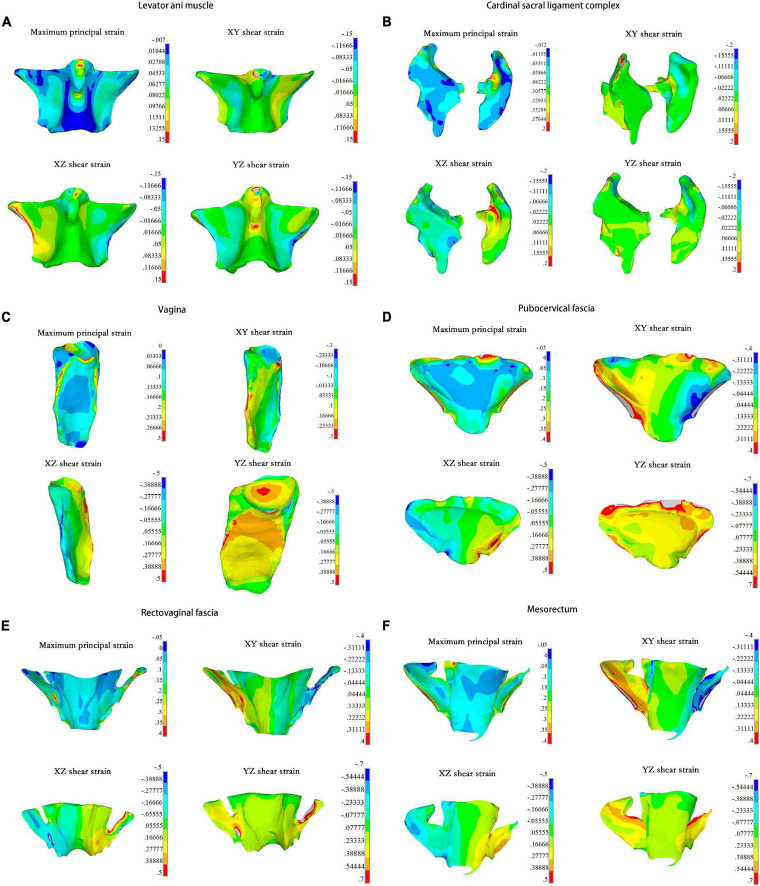
The maximum principal strain and strains of three directions in each pelvic organs under 0.01 MPa intra-abdominal pressure. For the **(A)** Levator ani muscle; **(B)** Cardinal sacral ligament complex; **(C)** Vagina; **(D)** Pubocervical fascia; **(E)** Rectovaginal fascia; **(F)** Mesorectum.

On the fascia, ligaments, and muscle, the compressive strength was higher than the tensile and shear strength, and the area with highest strain was more inclined to be injured. In our study, a set of elements with high strain in the pelvic support structure were selected to calculate the maximum principal and shear strains. The maximum positive principal strain showed that the levator ani muscle bore tension, and the maximum negative principal strain indicated that the levator ani muscle underwent compression. As shown in Figure 3A, the levator ani muscle bore tension in the middle of the front and both sides of the back. Higher tension and shear strains were detected from the junction of the levator ani muscle and obturator internus to the junction of the anterior levator ani muscle and pubic bone. Meanwhile, strain at the junction of the levator ani muscle and coccygeal muscle was relatively high, reaching more than 0.15.

The area with concentrated strain was detected at the upper connection between the right cardinal ligaments and the cervix, which had an amplitude of more than 0.2 (Figure 3B). The upper third of the vaginal lateral wall bore high tensile strain, while the lower parts bore high shear strain. The upper anterior vaginal wall and the top of the vagina also bore high shear strain, reaching 0.5 (Figure 3C). The tensile and shear strains at the junction of the pubocervical fascia and the obturator muscles were higher compared with other tissues, and the maximum shear strain in the YZ direction was more than 0.7 (Figure 3D). Similar results were found at the rectovaginal fascia and the side of the mesorectum, as shown in Figures 3E,F, respectively.

**FIGURE 4:**
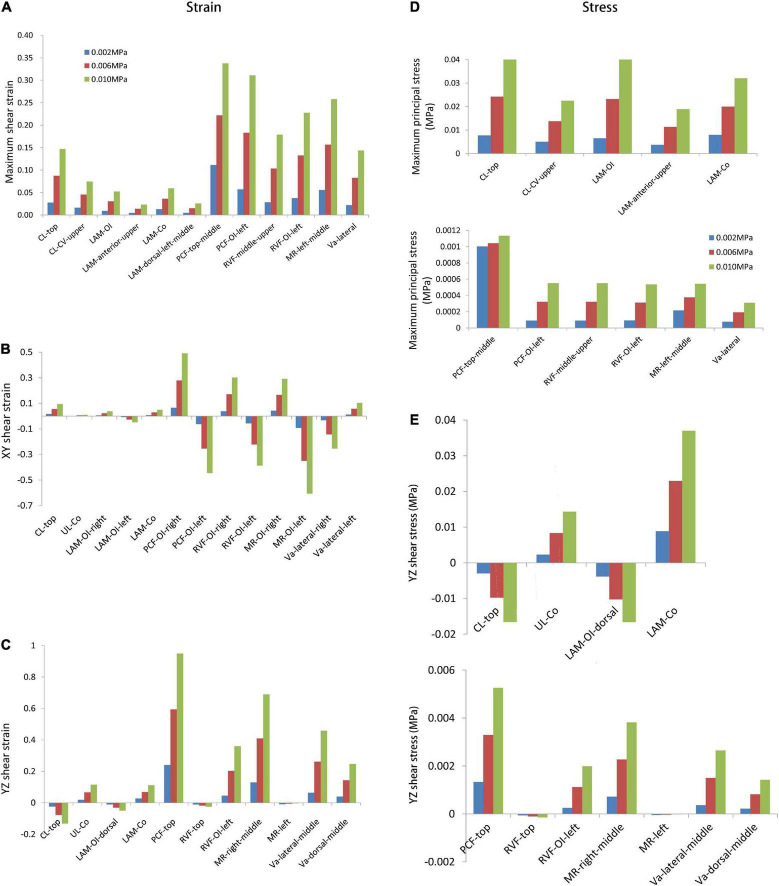
The maximum stress and strain values with intra-abdominal pressures ranging from 0.002 MPa to 0.01 MPa for the designated supporting tissues. The comparisons were shown for the **(A)** maximum shear strain; **(B)** XY shear strain; **(C)** YZ shear strain; **(D)** maximum principal stress; **(E)** YZ shear stress. CL, cardinal ligaments; CL-CV, cardinal ligaments-cervical junction; LAM, levator ani muscle; LAM-OI, levator ani muscle-obturator internus junction; LAM-Co, levator ani muscle-coccygeus junction; PCF, pubocervical fascia; PCF-OI, pubocervical fascia-obturator internus junction; RVF, rectovaginal fascia; RVF-OI, rectovaginal fascia-obturator internus junction; MR, mesorectum; MR-OI, mesorectum-obturator internus junction; UL-Co, uterosacral ligaments-coccygeus junction; Va, vagina.

The supporting tissue elements with high strain were selected to calculate the maximum principal strain and shear strain. The edge of the supporting tissues was not considered owing to the strain concentrations that may result from the calculation process. Figures 4A–C summarize the strain values with intra-abdominal pressures ranging from 0.002 to 0.01 MPa for each tissue. Strain values increased with increasing intra-abdominal pressure. The maximum principal strain was detected at the pubocervical fascia (Figure 4A), and the Y–Z shear strain reached 0.9 at the top of the pubocervical fascia (Figure 4C). A concentrated stress area was more likely at the levator ani muscle-obturator internus junction and cardinal ligaments-pelvic junction, which implied a high risk of clinical levator ani muscle and pelvic prolapse. The tension and shear strains for the lateral fascia were higher than those of the middle part connected with the organs. Thus, the probability of fascial and arcus tendineus fasciae pelvis prolapse was higher than that of a tear in the middle part connected with organs. This finding was also in agreement with the clinical result that there was a higher probability of displacement anterior wall prolapse than dilatation anterior wall prolapse.

Figures 4D,E show the maximum principle and shear stresses of the pelvic support structures, respectively. A similar trend was observed in that the maximum principle stress and shear stress increased with increasing intra-abdominal pressure. The maximum principle stress was 0.041 MPa at the top of the cardinal ligaments (Figure 4D), and the maximum shear stress was 0.037 MPa at the levator ani muscle-coccygeus junction (Figure 4E). These results indicated that the levator ani muscle and cardinal ligaments bore high tensile stress, as the maximum stress amplitude was very close between the levator ani muscle-obturator internus junction and the top of cardinal ligaments (Figure 4D). This was in line with clinical expectations and previous research results showed that the levator ani muscle and cardinal ligaments were the main support structures in load-bearing. The shear stress in the levator ani muscle was higher than that of the cardinal ligaments (Figure 4E).

## Discussion

**FIGURE 5:**
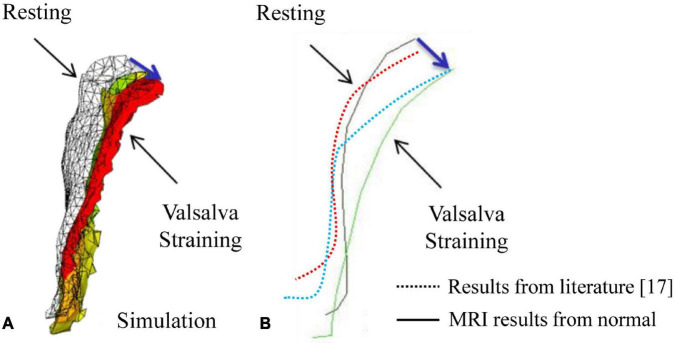
Displacement of the anterior vaginal wall during the Valsalva maneuver: **(A)** Results from our simulation; **(B)** Results from a previous study (12) and dynamic magnetic resonance imaging (MRI).

In this study, the compliance of the whole pelvic floor support system in a healthy female was studied using finite element analysis based on MRI. The vaginal wall displacement and the distributions of stress and strain in the supporting tissues were calculated under high intra-abdominal pressure. A similar displacement of anterior vaginal wall was observed between literature (11) and our study (5.58 mm for 100% pelvic floor muscle contraction under a downward pressure of 90 cm H_2_O vs. 5.29 mm under a uniform 100 cm H_2_O). Meanwhile, displacements of the vagina obtained in this study were in agreement with the data measured using clinical dynamic MRI. Figure 5 shows the displacement of the anterior vaginal wall compared with Larson et al.’s results reconstructed from dynamic MRI at rest and during the Valsalva maneuver (12). Our results and previous studies compare favorably. The results were also similar to those using dynamic MRI in asymptomatic volunteers (Figure 5B). All of these evaluation results confirmed the effectiveness of our study.

The posterior vaginal wall was more stable than the anterior vaginal wall in non-prolapsed women under high intra-abdominal pressure. In the vertical direction, the C point at the top was more stable than the anterior and posterior vaginal walls, while the stability of the C point was poorer in the anterior–posterior direction. According to our results, the anterior vaginal wall had the worst stability, which explained the high incidence of cystocele (13), clinically.

Previous studies showed that compliance varied in different vaginal regions (14). Compliance was the highest at the top of the vagina and lowest at the vaginal introitus. Our results were consistent with this observation and implied that non-prolapsed women also had compliance during high intra-abdominal pressure, and that vaginal wall movement should be restored intra-operatively, rather than be completely constrained (14). In a previous study (14), compliance in the top and bottom of the anterior vaginal wall was approximately 0.51 mm/cm H_2_O and 0.18 mm/cm H_2_O, respectively. The corresponding values were lower in our study, which was 0.048 and 0.09 mm/cm H_2_O, respectively. However, our results matched well with the dynamic MRI results of the volunteer during the Valsalva as well as physical examination in Peking University People’s Hospital. This discrepancy could be explained by two reasons: first, healthy women may vary in vaginal compliance; second, Spahlinger et al.’s research (14) was based on Caucasian anatomy, and our study was based on Asian anatomy. Different perineal body shapes and vaginal lengths could also lead to different compliance results.

Regarding strain in the vagina and supporting tissues, we found that strain at the sides of the levator ani muscle and pelvic fascia was higher, indicating that there were high risks of clinical prolapse between the levator ani muscle and arcus tendineus musculi levatoris ani and between the pelvic fascia and arcus tendineus fasciae pelvis. These areas were also susceptible to tear injuries, resulting in a higher risk of injury to the paravaginal supporting tissues. Previous studies reported that the incidence of paravaginal defects in patients with anterior vaginal wall prolapse was 38–80% (15, 16), and indicated that debonding of the pubocervical fascia and arcus tendineus fasciae pelvis clinically was the gold standard for diagnosing paravaginal defects (15). This study showed that both the levator ani muscle and the sides of the pelvic fascia were at a high risk of injury, and suggested that attention should be paid to lateral vaginal repair intraoperatively, which was also confirmed in Viana et al.’s study (17). The authors studied 66 women with symptomatic cystocele (grade 2–4) who underwent transvaginal paravaginal repair. Results showed that suspending the vesicovaginal fascia to the arcus tendineus fasciae pelvis was a safe and effective method for the treatment of paravaginal defects in patients with symptomatic cystocele, and the recurrence rate within 1 year was low (8.5%). The long-term effect of traditional anterior vaginal repair is poor, with a high recurrence rate of symptomatic cystocele.

In this study, we found that there was a high risk of injury at the top of the cardinal ligaments and cervix. High strains were also detected at the vaginal sidewall and the upper anterior vaginal wall, which may be related to the broadening of the vaginal wall. The results indicated that special attention should be paid to these regions in clinical evaluations, and comprehensive repair plans should be made to avoid complications and reduce the recurrence rate as much as possible.

The levator ani muscle and the cardinal sacral ligament complex bore high tensile and shear forces, which theoretically proved that the pelvic floor played an important role in the supporting tissues. This result was consistent with the finding by Chen et al. (4). The levator ani muscle bore high Y–Z shear stress to prevent the pelvic viscera from excessive downward movement. According to previous studies, the levator ani muscle has fiber orientation (18). It holds a strong load-bearing capacity along the horizontal fiber direction, while its vertical fiber direction cannot bear excessive load. The distribution of fibers in the levator ani muscle was from one side of the obturator internus to the other side of the obturator internus (19), which was the X direction in our model. Therefore, the levator ani muscle could not bear excessive Y–Z shear load, and the side of the levator ani muscle and the back area connected with the coccygeal muscle was more susceptible to injury.

Our results showed that the pelvic floor support system in a healthy female was sensitive to increasing intra-abdominal pressure. Chronic high intra-abdominal pressure, such as with obesity, chronic cough, and chronic constipation are risk factors for POP (20, 21). Avoiding high intra-abdominal pressure exercises as well as training to increase pelvic floor muscle strength could reduce the risk of pelvic floor support tissue injury and prevent POP (22).

Regarding the boundary conditions in the finite element analysis, previous studies applied the load perpendicular to the vaginal sidewall to simulate the pelvic system during high intra-abdominal pressure. Chen et al. (4) applied a perpendicular pressure to the surface of the anterior vaginal wall, and Luo et al. (6) applied the pressure perpendicular to the nodes on the anterior and posterior vaginal wall, perineal body, and levator ani muscle. However, accurate loads on the vaginal wall surface are difficult to estimate (23), and the anterior vaginal wall *in vivo* is connected with the pubocervical fascia, not directly exposed to the abdominal cavity. Thus, the simulated perpendicular pressure on the vaginal wall surface differs from the actual conditions *in vivo*. Chen et al. (23) introduced a new idea in displacement loading by applying a specific displacement on the top of the uterus to simulate intra-abdominal pressure. However, applying displacement constraint only on the top of the uterus leads to inaccurate stress distributions in other organs that are not loaded because intra-abdominal pressure is transmitted to the surface of organs through the peritoneum *in vivo*. In the current study, we first established a peritoneal structure, and then applied uniform intra-abdominal pressure on its surface. The pressure was transmitted to the surface of the pelvic organs through the connective tissues, which more closely simulated natural pelvic loading.

There are limitations that should be acknowledged. First, although the isotropic linear elastic material parameters were derived from previous studies, the material properties may vary with different methods and different measurement conditions. Furthermore, some tissues, such as the perineal body, ligamentous complex, and fascia, exhibited viscoelastic properties; however, we did not consider the effect of a load that changes over time. Second, this study focused only on the passive stretching of the levator ani muscle, as the intra-abdominal pressure was applied by the volunteer under the condition of levator ani muscle relaxation. Anisotropy, hyperelasticity, and active contractility of the levator ani muscle were not taken into account.

## Conclusion

In conclusion, this is the first study to investigate the distributions of strain and stress as well as the high-risk injury areas in the whole pelvic floor support system. Based on the biomechanical characteristics of healthy women, our results showed that the levator ani muscle, the sidewall of the pelvic fascia, proximal of the cardinal ligaments, vaginal sidewall, and the upper anterior vaginal wall were vulnerable to injury due to the development of stress concentrations. These findings can be used to evaluate the potential injury areas in non-prolapsed women under high intra-abdominal pressure. It is also suggested that comprehensive clinical repair plans should be made to reduce post-operative complications and recurrence rates.

## Data availability statement

The raw data supporting the conclusions of this article will be made available by the authors, without undue reservation.

## Ethics statement

The studies involving human participants were reviewed and approved by the Ethics Committee at Peking University People’s Hospital (Reference Number: IRB00001052-18018). The patients/participants provided their written informed consent to participate in this study. Written informed consent was obtained from the individual(s) for the publication of any potentially identifiable images or data included in this article.

## Author contributions

QR and SR contributed to the conception of the study. JW, XL, and BX performed the clinical experiment. SR contributed significantly to finite element analysis and data analyses. XL performed the manuscript preparation and wrote the manuscript. YL, QR, SR, and XL helped perform the analysis with constructive discussions. All authors contributed to the article and approved the submitted version.
